# STAT6 expression in glioblastoma promotes invasive growth

**DOI:** 10.1186/1471-2407-11-184

**Published:** 2011-05-20

**Authors:** Barbara C Merk, Jennifer L Owens, Maria-Beatriz S Lopes, Corinne M Silva, Isa M Hussaini

**Affiliations:** 1 Department of Pathology, University of Virginia, Charlottesville, VA 22908, USA; 2 Department of Microbiology, University of Virginia Charlottesville, VA 22908, USA; 3 Department of Molecular Physiology and Biological Physics, University of Virginia, Charlottesville, VA, 22908, USA

## Abstract

**Background:**

Glioblastoma (GBM) is a highly aggressive malignant primary brain tumor, characterized by rapid growth, diffuse infiltration of cells into both adjacent and remote brain regions, and a generalized resistance to currently available treatment modalities. Recent reports in the literature suggest that Signal Transducers and Activators of Transcription (STATs) play important roles in the regulation of GBM pathophysiology.

**Methods:**

STAT6 protein expression was analyzed by Western blotting in GBM cell lines and by immunohistochemistry in a tissue microarray (TMA) of glioma patient tissues. We utilized shRNA against STAT6 to investigate the effects of prolonged STAT6 depletion on the growth and invasion of two STAT6-positive GBM cell lines. Cell proliferation was assessed by measuring ^3^H-Thymidine uptake over time. Invasion was measured using an *in vitro *transwell assay in which cells invade through a type IV collagen matrix toward a chemoattractant (Fetal Bovine Serum). Cells were then stained and counted. Kaplan-Meyer survival curves were generated to show the correlation between STAT6 gene expression and patient survival in 343 glioma patients and in a subset of patients with only GBM. Gene expression microarray and clinical data were acquired from the Rembrandt [1] public data depository (https://caintegrator.nci.nih.gov/rembrandt/). Lastly, a genome-wide expression microarray analysis was performed to compare gene expression in wild-type GBM cells to expression in stable STAT6 knockdown clones.

**Results:**

STAT6 was expressed in 2 GBM cell lines, U-1242MG and U-87MG, and in normal astrocytes (NHA) but not in the U-251MG GBM cell line. In our TMA study, STAT6 immunostaining was visible in the majority of astrocytomas of all grades (I-IV) but not in normal brain tissue. In positive cells, STAT6 was localized exclusively in the nuclei over 95% of the time.

STAT6-deficient GBM cells showed a reduction in ^3^H-Thymidine uptake compared to the wild-type. There was some variation among the different shRNA- silenced clones, but all had a reduction in ^3^H-Thymidine uptake ranging from 35%- 70% in U-1242MG and 40- 50% in U-87MG cells. Additionally, STAT6- depleted cells were less invasive than controls in our *in vitro *transmembrane invasion assay. Invasiveness was decreased by 25-40% and 30-75% in U-1242MG and U-87MG cells, respectively. The microarray analysis identified matrix metalloproteinase 1 (MMP-1) and urokinase Plasminogen activator (uPA) as potential STA6 target genes involved in the promotion of GBM cell invasion. In a Kaplan-Meier survival curve based on Rembrandt [1] gene expression microarray and clinical data, there was a significant difference in survival (P < 0.05) between glioma patients with up- and down-regulated STAT6. Decreased STAT6 expression correlated with longer survival times. In two subsets of patients with either grade IV tumors (GBM) or Grade II/III astrocytomas, there was a similar trend that however did not reach statistical significance.

**Conclusions:**

Taken together, these findings suggest a role for STAT6 in enhancing cell proliferation and invasion in GBM, which may explain why up-regulation of STAT6 correlates with shorter survival times in glioma patients. This report thus identifies STAT6 as a new and potentially promising therapeutic target.

## Background

Each year, roughly 18,000 new cases of malignant primary brain tumors are diagnosed in the United States, the majority of which are gliomas. Of these, 50-60% are classified as World Health Organization grade IV astrocytomas, or Glioblastomas (GBM) [2], which makes GBM the most common primary brain tumor in adults. GBM is also the most aggressive and most lethal type of brain tumor, with an average patient life expectancy of only 15 months after diagnosis [3]. GBM cells are not only highly proliferative but also readily invade surrounding brain structures, thereby making complete surgical resection practically impossible [4]. Furthermore, the majority of GBMs are intrinsically resistant to most forms of radio- and chemotherapy [5,6], thus rendering the standard arsenal of anti-cancer treatments rather ineffective. The relatively recent addition of temozolomide to standard treatment regimens consisting of surgical resection and radiation extended median survival time from 12.1 to 14.6 months and more than doubled overall 2-year survival from 10.4 percent to 26.5 percent [7]. While these therapeutic advances are encouraging, there is clearly still a dire need for more effective therapeutic approaches. A better understanding of the mechanisms controlling the GBM phenotype is essential for the identification of new molecular targets.

The Signal Transducers and Activators of Transcription (STAT) family of transcription factors consists of seven members (STATs 1-4, 5a, 5b, and 6), several of which possess properties of oncogenes. STAT3 for instance, is up-regulated and active in breast, prostate, lung, head and neck, pancreatic and colon cancer as well as melanoma, leukemia and lymphoma [8-15]. Recently, STAT3 was reported to be over expressed and active in gliomas, and its deletion induces spontaneous apoptosis in glioma cell lines [16-18]. STAT5b appears to play an important role in several aspects of GBM pathophysiology, as was shown by Liang *et al*. who demonstrated its involvement in glioma cell proliferation, cell cycle progression, and invasion [19].

Despite the fact that each STAT family member responds to distinct stimuli, resulting in a specific cellular response, all STATs share a similar mechanism of activation and function [9]. STAT activity is initiated by phosphorylation of a conserved tyrosine residue near the C terminus, most commonly by Janus Kinases (JAKs). Receptor tyrosine kinases such as the epidermal growth factor receptor (EGFR) and platelet-derived growth factor receptor (PDGFR), as well as non-receptor tyrosine kinases (i.e. v-Src, v-Abl) can also phosphorylate STAT proteins [20,21].

Tyrosine phosphorylated STATs form dimers and translocate to the nucleus, where they bind their target DNA sequence, recruit co-activators and initiate transcription of target genes. Over 100 potential STAT target genes have been identified [21], many of which are involved in the control of cell proliferation, differentiation, and apoptosis [22]. Altered expression of these genes has been linked to cellular transformation and oncogenesis [9]. Specifically, STATs 3 and 5b induce members of the Bcl-2 family of anti-apoptotic regulatory proteins [23,19] as well as cyclin D1, which promotes cell-cycle progression [24,25]. Additionally, STAT3 regulates the expression of the c-Myc transcription factor, which facilitates cell proliferation and survival and is frequently over-expressed in human cancers [24,26,27].

In non-transformed cells, STAT signaling is transient and results from the activation of specific pathways. Constitutive activation of STATs has, however, been demonstrated in several human malignancies including breast, lung, prostate, pancreatic and renal cancer, as well as several types of leukemia and lymphoma [9]. The activation of STATs in transformed cells is generally achieved by over-activity of tyrosine kinases, either due to an activating mutation in the kinase itself, or as a result of increased signaling by cytokines and growth factors. In breast cancer, for instance, increased STAT activity is a consequence of excessive signaling of the EGFR pathway and c-src [9]. These aberrantly activated STATs can render the cell independent of cytokine- or growth factor-induced signals, while simultaneously altering the normal gene expression pattern in favor of growth and survival.

Compared with other STAT family members, the involvement of STAT6 in human cancer has received limited attention. Nevertheless, STAT6 is over-expressed and active in numerous malignancies including prostate and colon cancer [28,29], lymphoma [30-32], and leukemia [33,34]. Furthermore, STAT6 has been implicated in the prevention of apoptosis in human colon cancer cells [35], and its expression in these cells positively correlates with increased invasive and metastatic capabilities [29].

In this study, we investigated the involvement of STAT6 in GBM proliferation and invasion. First, we showed robust STAT6 expression in 2 of 3 GBM cell lines. In a tissue microarray (TMA) of human glioma patients, glioma tissue specimens consistently exhibited higher STAT6 levels than did non-malignant brain tissue. Expression levels however did not appear to correlate with tumor grade. We further demonstrated that in at least one GBM cell line, STAT6 exhibited basal activity in the absence of external stimuli- an observation that agrees with the predominantly nuclear localization seen in immunohistochemistry of human glioma tissues. Additionally, STAT6 was activated by relevant signalling molecules *in vitro*, including epidermal growth factor (EGF), whose receptor is frequently up-regulated/amplified in GBM and correlates with shorter survival times in patients. Kaplan-Meier survival curves generated with Rembrandt [1] -derived patient data also showed a correlation between higher STAT6 expression and decreased survival of glioma patients. Finally, GBM cells in which STAT6 had been silenced with shRNA exhibited markedly decreased rates of proliferation and invasion compared with wild-type GBM cells. A gene expression microarray identified MMP-1 and uPA as potential STAT6 target genes and downstream modulators of cell invasion.

## Methods

### Reagents

EGF was purchased from Chemicon/Millipore (Billerica, MA). The tissue micro array (TMA), the antibody against STAT6 used for Immunohistochemistry (Ab641) and the phospho-STAT6 (Tyr641) antibody were purchased from Imgenex Corp. (San Diego, CA). Rabbit polyclonal antibodies against STAT5a and STAT6 used for Western blotting were purchased from Santa Cruz Biotechnology, Inc. (Santa Cruz, CA). Rabbit polyclonal antibodies against STAT1, STAT2, STAT3 and STAT4 were purchased from Cell Signaling Technology (Beverly, MA). The antibody against STAT5b was a generous gift from Dr. C. Silva (University of Virginia). The mouse monoclonal α-tubulin antibody (Product Number T6074), MISSION shRNA Lentiviral Transduction Particles against STAT6 (Product Number NM_003153) and MISSION Non-Target shRNA Control Transduction Particles (Product Number SHC002V) were purchased from Sigma-Aldrich (St. Louis, MO). The HG-U133_Plus_2 gene chip was purchased from Affymetrix (Santa Clara, CA).

### Cell Culture

The U-1242MG and U-251MG cell lines were generously supplied by Dr. A.J. Yates (Ohio State University) and Dr. DD Bigner (Duke University), respectively. Both cell lines were isolated from characterized GBM tumors and have been extensively described elsewhere [36]. The U-87MG cell line was obtained from American Type Culture Collection (Manassas, VA). Cells were cultured in minimal essential medium-α (MEM- α) supplemented with 10% fetal bovine serum (FBS) (Hyclone, Logan, Utah) and 1% penicillin/streptomycin (Invitrogen) at 37°C in 4.8% CO_2_, 90% relative humidity unless stated otherwise.

Primary cultures of human fetal astrocytes (NHA) were obtained from Clonetics (San Diego, California) and cultured in a growth medium containing 25 μg/ml bovine insulin, 20 ng/ml EGF, 5% fetal bovine serum, 20 ng/ml progesterone, and 50 μg/ml transferrin at 37°C in 4.8% CO_2_, 90% relative humidity.

### Western blot analysis

Cells were rinsed with 1x phosphate buffered saline (PBS; 137 mM NaCl, 8.1 mM Na_2_HPO_4_, 2.7 mM KCl, and 1.5 mM KH_2_PO_4_, pH 7.4) containing 0.2 mM sodium orthovanadate and protein was extracted using Triton lysis buffer [1% Triton X-100, 50 mM Tris, pH 7.5, 150 mM NaCl, 2 mM EDTA, and Protease Inhibitor Cocktail (Sigma-Aldrich, Product Number P8340)] additionally containing 2 mg/ml sodium orthovanadate (phosphorylation studies only) and 5 mg/mL DTT unless otherwise noted. Western blot analysis was performed as previously described [36].

### RNA extraction

Cells were grown to 90% confluence in 100 mm plates in MEM- α medium with 10% FBS and 1% penicillin/streptomycin. Each dish was lysed at room temperature by applying 1 ml of Trizol reagent (Invitrogen) and gently pipetting up and down until all cells were suspended in the solution. Lysates were combined with 200 μl of chloroform in RNAse/DNAse free 1.5 ml centrifuge tubes and centrifuged at 14,000 × g for 15 minutes. Upon removal from the centrifuge, the mixture consisted of two layers; the top layer containing the RNA was carefully transferred into a new 1.5 ml centrifuge tube and combined with 500 μl of isopropanol at - 20°C overnight to facilitate RNA precipitation. The next day, RNA was pelleted by centrifugation at 14,000 × g for 10 minutes. The supernatant was removed, and the RNA pellet was washed once by adding 1 ml of 75% ethanol followed by centrifugation at 8,000 × g for 5 minutes. The ethanol was removed, and the pellet was allowed to dry in the open tube for about 10-15 minutes depending on pellet size. The dry pellet was then re-suspended in RNAse free/DEPC water (10-20 μl depending on pellet size) and concentration was determined by spectrophotometer.

### Real-time (quantitative) PCR

Primers were designed using Primer Express 2.0 (Applied Biosystems), based on target sequences retrieved from the Affymetrix Probe Sequence Database (Liu et al., 2002). Total RNA samples were prepared as described above. Reverse transcription PCR was performed using MultiScribe reverse transcriptase (Applied Biosystems, Forster City, CA) and random hexamers as per the manufacturer's instruction, to generate cDNAs. Real time quantitative PCR using SYBR Green I was then performed on the cDNAs in an Applied Biosystems 7900 Sequence Detection System. Samples were run in triplicate. In order to verify that only a single PCR product was amplified per transcript, dissociation curve data was analyzed through the 7900HT Sequence Detection Software (SDS). To account for differences in starting material, quantitative PCR was also carried out for each cDNA sample using housekeeping genes synthesized at our own facility, hypoxanthine-guanine phosphoribosyltransferase (HPRT) and β-actin. The data collected from these quantitative PCRs defined a threshold cycle (Ct) of detection for the target or the housekeeping genes in each cDNA sample. Analysis of the variance (ANOVA) was then performed to determine the mean and standard error for each comparison.

### shRNA gene silencing

U-1242 MG and U-87MG cells were seeded in 6-well plates and grown to 60% confluence in MEM- α medium with 10% FBS, at 37°C in 4.8% CO_2_, 90% relative humidity Six wells of each cell line were then transduced with one of five MISSION lentiviral shRNA transduction particles targeting STAT6 or with a control (non-targeting) shRNA, according to manufacturer protocol (Titer: 10^6 TU). The vector for all shRNAs was pLKO.1; the five STAT-6 targeting sequences were as follows:

TRCN0000019409: CCGGGCAGGAACATACAGACACATTCTCGAGAATGTG TCTGTATGTTCCTGCTTTTT

TRCN0000019410: CCGGGCCTTCTTATGACCTTGGAATCTCGAGATTCCAA GGTCATAAGAAGGCTTTTT

TRCN0000019411: CCGGGCTTGATAGAAACTCCTGCTACTCGAGTAGCAG GAGTTTCTATCAAGCTTTTT

TRCN0000019412: CCGGGCCACTTTCAGACAAATACTTCTCGAGAAGTATT TGTCTGAAAGTGGCTTTTT

TRCN0000019413: CCGGGTCGCAGTTCAACAAGGAGATCTCGAGATCTCCT TGTTGAACTGCGACTTTTT

48 hours after transduction, 1.5 μg/ml puromycin was added to each well. Cells were selected for resistance for 10 days, after which the mixed culture was screened for STAT6 expression by Western blot analysis. Each sample was also screened for off-target effects on STATs 3, 5a and 5b at this time. These three STATs were chosen due to their documented importance in GBM in the literature [16-19,39,40]. Mixed cultures displaying the best knockdown of STAT6 in combination with the fewest off-target effects were subsequently subjected to dilution cloning: cells from the mixed cultures were plated at a density of one cell per well of a 96-well plate, and each clone was expanded and screened for STAT6 expression by Western blot analysis. For U-87MG, TRCN0000019409 and TRCN0000019413 were the two sequences with the best results; for U-1242MG it was TRCN0000019411 and TRCN0000019413. Clones derived from each sequence were named accordingly; for example, U-1242MG clone **11**:22 was originally transduced with sequence TRCN00000194**11**, while U-87MG clone **13**:38 was transduced with sequence TRCN00000194**13**.

### ^3^H-Thymidine Incorporation

The relative rate of cell proliferation was determined by the measurement of ^3^H-thymidine incorporation into DNA, as previously described [36]. Briefly, cells were counted and plated in 24-well plates at a density of 1.5×10^4 ^cells/well (U-1242MG) or 5×10^5 ^cell/well (U-87MG). Cells were allowed to grow for 72 h in MEM- α medium supplemented with 10% FBS and 1% penicillin/streptomycin (Invitrogen) at 37°C in 4.8% CO_2_, 90% relative humidity, then pulsed with ^3^H-thymidine (1 μCi/mL) for 4 h. Cells were washed 3× with 1 ml/well cold 1x PBS, fixed with 1 ml/well of 10% trichloroacetic acid (TCA) for 10 minutes on ice, washed 3x with room-temperature PBS (1 ml/well), and permeabilized in 1 ml/well 1N NaOH overnight at room temperature. The pH was then neutralized with an equal volume (1 ml/well) of 1 M HCl and the solution was transferred into scintillation vials containing Ready-Safe scintillation fluid (10 mL). A Beckman Liquid Scintillation Counter was used to quantify ^3^H-thymidine uptake by the cells. All samples were run in triplicate, and each assay was repeated three times.

### In vitro Invasion Assay

Invasion was determined using a variation of the Boyden chamber assay, as described in [37]. Briefly, cells were trypsinized and counted; next, 5 × 10^5 ^cells (U-87MG) or 1.5 × 10^4 ^cells (U-1242MG) were suspended in 300 μl of either serum-free MEM-α (U-1242MG) or MEM-α containing 0.1% FBS (U-87MG). The cells were seeded into the upper compartment of a Type IV collagen (Sigma) coated polycarbonate filter with a pore size of 8.0 μm (Becton Dickinson) in a 24-well plate. Each polycarbonate filter had been coated with 10 μl of 30% Type IV collagen 24 h before the addition of cells. 500 μl MEM-α medium containing 10% FBS was added to the lower compartment as a chemo attractant. After 8 h of incubation at 37°C in 4.8% CO_2_, 90% relative humidity, filters were fixed and stained: the medium was removed from the top and bottom chambers and replaced with a 0.1% crystal violet stain for 1 minute at room temperature. The filters were then gently rinsed with de-ionized water to remove excess crystal violet. Cells in the upper compartment were removed, leaving only the cells on the underside of the filter- these represented those cells who had successfully invaded across the collagen-coated filter. Cells were photographed under a LEICA DMIRE 2 microscope using a QImaging RETIGA EXi digital camera. The entire visual fields were photographed, and the cells were counted. All samples were run in triplicate, and assays were repeated at least twice.

### Tissue Microarray and Immunohistochemical Staining

The Tissue Microarray was purchased from Imgenex (San Diego, CA). It included tissue sections from 8 patients with WHO Grade IV astrocytoma (GBM), 5 patients with Grade III (anaplastic) astrocytoma, 17 patients with Grade II (diffuse) astrocytoma, 8 patients with Grade I (pilocytic) astrocytoma. It also included 8 sections of normal brain tissue.

Slides were deparaffinized in xylene and rehydrated in ethanol according to manufacturer protocol. Immunostaining was performed using a STAT6 primary antibody.

Two independent investigators visually classified each tissue sample as either STAT6 positive or negative. It should be noted that STAT6 was frequently and highly expressed in vascular endothelial cells surrounding blood vessels seen in the specimens; however a designation of positive or negative was used to refer exclusively to STAT6 expression in tumor cells.

### Statistical Analysis

The mean and standard error of the mean (S.E.M.) were calculated for each triplicate point by using Prism VI (Graphpad Software, San Diego, CA, USA), and error bars represent the S.E.M.. Each experiment was performed a minimum of three times. Numerical values of each separate run were normalized against the Non-Target Control to generate the graphs (Figures 5 and 6). Statistical significance was calculated via One-way ANOVA followed by Dunnett's Multiple Comparison Test, in reference to the Non-Target Control rather than the wild type. However, all samples labeled with an * were also significantly different from the wild type in the same analysis. The level of significance was taken at P < 0.05 at a confidence interval of 95%.

### Kaplan-Meier Survival Plot

#### Ethics Statement

All human subjects data was publicly available in de-identified form on the Rembrandt website (https://caintegrator.nci.nih.gov/rembrandt/). Therefore, its use was not classified as human subjects research, and no Institutional Review Board approval was needed.

### Patient Datasets and Data Analysis

Both the microarray gene expression data and the clinical data were obtained from the NCI Repository for Molecular Brain Neoplasia Data (REMBRANDT) database (https://caintegrator.nci.nih.gov/rembrandt/) [1], using data available on October 1^st^, 2010. The clinical data were originally obtained from contributing institutions including the Henry Ford Hospital, UCSF, Lee Moffitt Cancer Center, Dana Farber Cancer Center, University of Wisconsin, and NCI. Diagnoses were also made at the respective clinics. At the time of access, 343 glioma patient samples with both gene expression data and corresponding survival times were available on the Rembrandt database. These included 181 GBMs, 105 grade II/III astrocytomas, 50 grade II/III oligodendrogliomas and 7 mixed gliomas.

Three Kaplan-Meier survival curves were generated: one using available data on all glioma patients (n = 343), another looking at GBM patients only (n = 181), or only using data on Grade II/III astrocytoma patients (n = 105). The graphs were created using Rembrandt microarray data for the probes from the Affymetrix U133 Plus 2.0 GeneChip and associated survival data. The "Highest Geometric Mean Intensity" of STAT6 was used as the reporter for relative STAT6 expression within the database. STAT6 up- or down-regulation was defined as a 2-fold (or greater) difference from the mean expression level within a given data set. For example, up-regulation among GBM patients refers to a 2-fold (or greater) increase in STAT6 expression, compared to the average STAT6 expression levels in all patients within the GBM sub-population. Therefore, each patient sub-population (GBM, astrocytoma, or all gliomas) has a distinct baseline, and individual patients' STAT6 expression levels are only compared to other patients in the same sub-population.

### Affymetrix microarray

Microarray analysis of Affymetrix chips was performed as previously described in [38]. Briefly, total RNA was extracted from wild type and STAT6-deficient U-1242MG and U-87MG cells. Biotin-labeled cRNA was prepared from approximately 2 ug of total RNA and hybridized to Human Genome U133-plus 2 (HG-U133_Plus_2) Affymetrix oligonucleotide arrays, which contain approximately 56,400 transcripts of human genes or ESTs. After washing in a fluidic station, the arrays were scanned with a 2.5-micron resolution Affymetrix Microarray Scanner (Affymetrix, Santa Clara, CA). Scanned images were first examined for visible defects and then checked for fitness of the gritting. The image file was then analyzed to generate a raw data file. From this point on a coordination of two paths of analysis was carried out using Affymetrix Microarray Analysis Suite 5.0 (MAS 5.0, Affymetrix, Santa Clara, CA) and Dchip software [39]. The detection of a particular gene, called "present", "absent", or "marginal", was made using the nonparametric Wilcoxon ranked score algorithm as provided in MAS 5.0; those detection calls were then imported into and utilized by the Dchip program. Scatter plots were also generated using this software to inspect the reproducibility of the replicates as well as the degree of variations of the samples under comparison. Quantitation of the genes was performed using Dchip, which applied a model-based approach to derive the probe sensitivity index and expression index. The two indices were used in a linear regression to quantify a particular gene. When specific probes or transcripts deviated from the model to a set extent, they were identified as outliers and thus excluded from the quantitation process. Normalization of the arrays was performed using the invariant set approach. Comparative analysis of the samples using Dchip generated fold changes and paired sample t-test p-values. We considered a p < = 0.05 and a fold change > = 1.5 in combination of a % Present > = 50 as an indication of significant change in gene expression for up-regulation or down-regulation. A Spearman correlation coefficient was generated for all possible pairs involved using the Dchip quantitation results for quality control. Hierarchical clustering of the genes was performed after an appropriate filtration of the data.

## Results

### STAT6 is expressed in GBM cell lines and patient astrocytoma specimens

It has been reported by others [40,19] that STATs 3 and 5 are expressed in GBM, where they perform numerous oncogenic functions. Specifically, high STAT3 expression contributes to cell cycle progression, survival, and immune evasion in GBM [18,41-43], while STAT5 facilitates GBM cell proliferation and invasion [19]. Rahaman *et al*. [44] showed that STAT6 is also expressed in GBM cell lines.

**Figure 1:**
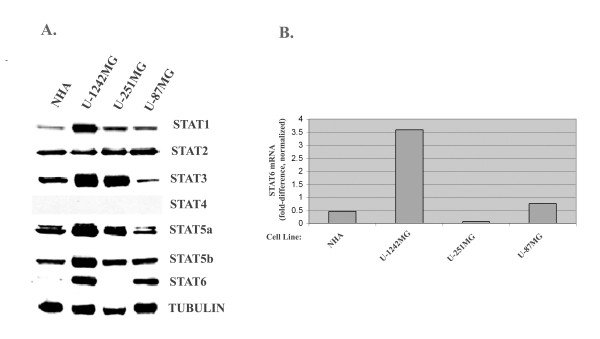
**STAT6 Expression in GBM cells**. **Figure 1a**: Western blot comparing expression of STATs 1-6 in NHA and three GBM cell lines. STATs 1, 3, 5a, 5b and 6 are over-expressed in at least one GBM cell line compared to the non-malignant astrocytes. STAT4 was not expressed in GBM or NHA. **Figure 1b**: Real-time (quantitative) PCR showing increased STAT6 mRNA in 2 of 3 GBM cell lines compared to NHA. mRNA expression was normalized by the geometric mean of the housekeeping genes beta actin and HPRT.

In order to establish the expression profiles of STATs in GBM, we examined protein expression levels of all seven STATs by Western blot analysis in three GBM cell lines (U-1242 MG, U-87 MG and U-251 MG) and compared them to expression levels in non-malignant fetal astrocytes ("normal human astrocytes", NHAs) (Figure 1A). Not surprisingly, STATs 3, 5a and 5b were each up-regulated in at least one GBM cell line compared with NHAs, confirming earlier reports in the literature [19,42]. STAT6 protein expression was markedly increased in two of the three GBM cell lines (U-1242 MG and U-87MG) when compared with the NHAs. Alpha-tubulin was used as the loading control.

Next, we wanted to assess whether increased STAT6 protein levels in GBM cells were a direct consequence of elevated mRNA levels, or if they were primarily a result of slower protein turnover. We therefore examined STAT6 mRNA levels in each cell line by real-time (quantitative) PCR. Figure 1b shows relative levels of STAT6 mRNA in NHAs, U-1242MG, U-251MG and U-87 MG cell lines, normalized to the housekeeping genes hypoxanthine-guanine phosphoribosyltransferase (HPRT) and β-actin. In U-1242MG cells, mRNA for STAT6 was increased more than 7-fold compared with NHAs, and was also much higher than in the other two GBM cell lines (Figure 1B). U-87MG cells also had increased STAT6 mRNA levels compared with the control; however, this was a more modest increase of only about 50%. The mRNA expression pattern of STAT6 in the four cell lines therefore generally agrees with STAT6 protein expression levels, which also were increased in U-1242MG and U-87MG, but not in U-251MG cells when compared with NHAs. Nevertheless, the 4-fold difference in STAT6 mRNA between U-1242MG and U-87MG was not apparent at the protein level.

Taken together, these results suggest that an increase in mRNA levels likely contributes to the increased expression of STAT6 seen at the protein level. Whether the elevated transcript levels are due to increased transcription or improved mRNA stabilization remains to be determined. Additionally, it is possible that protein turnover of STAT6 in GBM cells is abnormal as well, which would explain the high STAT6 protein levels in U-87MG cells in the absence of a corresponding increase in the transcript.

### STAT6 is expressed in gliomas of Grades I-IV, but not in normal cortex

**Figure 2:**
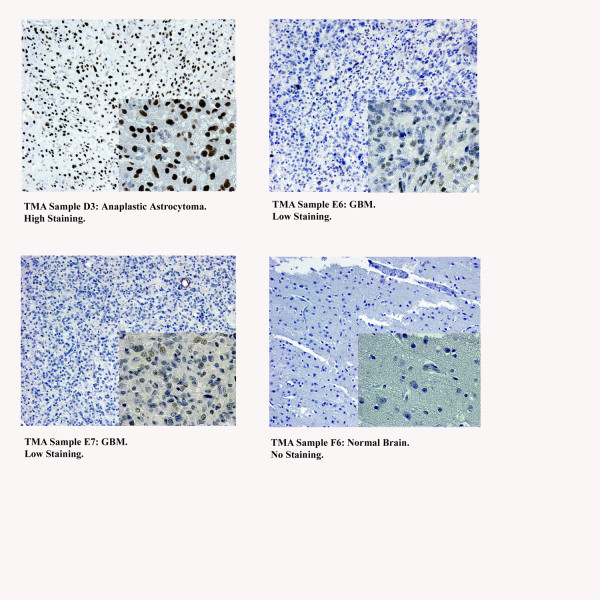
**Tissue Microarray (TMA), representative images of staining categories**. All images were probed for total STAT6 protein by IHC. Upper left: tissue sample of a Grade III (anaplastic) astrocytoma showing high staining intensity in the nuclei of the majority of cells. Upper right: GBM tissue sample demonstrating an example of low staining: some nuclei exhibit dark staining, but less than half the cells are positive for STAT6. Lower left: GBM sample showing weak staining intensity in the majority of nuclei. This was also called low staining. Lower right: Tissue sample of normal brain, showing no STAT6 staining in the nucleus or elsewhere.

In order to relate our *in vitro *findings to actual human patient tumor specimens, we utilized a tissue microarray (TMA) to evaluate STAT6 expression in GBM, healthy brain, and lower grade gliomas by immunohistochemistry (IHC). Two independent investigators examined 8 sections each of normal cortex, Grade I (pilocytic) astrocytoma, and Grade IV astrocytoma (GBM), as well as 5 sections of Grade III (anaplastic) astrocytoma and 17 sections of Grade II (diffuse) astrocytoma, and evaluated the extent and intensity of STAT6 staining in each sample. Figure 2 shows examples of images from the TMA, and the numerical results of all TMA sections are summarized in Table 1. Tumor-associated endothelial cells, which frequently displayed high intensity staining of STAT6, were disregarded when describing a sample as STAT6 positive or negative.

**Table 1:** Comparison of STAT6 expression between Grades I-IV astrocytomas and normal brain on the TMA.

		STAT6 Expression (%)	
		
N		Negative	Positive
Normal Brain	8	8 (100%)	0

Pilocytic Astrocytoma (Grade I)	8	3 (37.5%)	5 (62.5%)

Diffuse Astrocytoma (Grade II)	17	3 (17.6%)	14 (82.4%)

Anaplastic Astrocytoma (Grade III)	5	0	5 (100%)

Glioblastoma (Grade IV)	6	1 (16.6%)	5 (83.4%)

No STAT6 staining was seen in the 8 sections of normal cortex. It is, however, likely that expression levels were simply too low to be detectable by IHC in our study, given previous reports of STAT6 expression in astrocytes [44] and our own findings that STAT6 is expressed, albeit at low levels, in NHAs.

STAT6 staining was observed in 5 of 8 (62.5%) pilocytic astrocytomas (Grade I), 14 of 17 (82.3%) diffuse astrocytomas (Grade II), 5 of 5 (100%) anaplastic astrocytomas (Grade III) and 4 of 5 (83.4%) GBM. There does not appear to be a correlation between STAT6 expression and tumor grade, suggesting STAT6 may play a role early in the process of transformation. The fact that STAT6 over-expression is consistently maintained in high-grade astrocytomas does imply possible additional functions for STAT6, potentially involving tumor maintenance and/or progression.

### EGF induces STAT6 tyrosine phosphorylation in vitro

It is generally accepted that STATs are phosphorylated in response to growth factor signaling in a variety of cancer cell lines [9]. The EGF receptor (EGFR) is frequently amplified, over-expressed or mutated in GBM where it plays a vital role in tumor development and maintenance [5]. Increased EGFR expression and activity- both as a response to external stimuli or due to a gain-of-function mutation- correlate with an exceptionally poor prognosis in human GBM patients.

**Figure 3:**
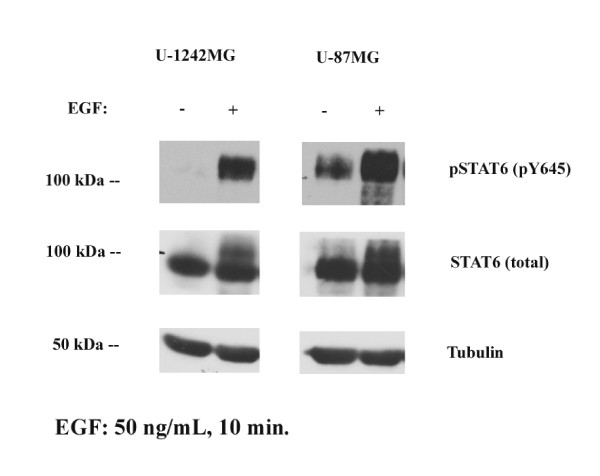
**STAT6 activation by EGF**. The GBM cell lines U-1242MG and U-87MG were serum-starved for 12h followed by treatment with EGF (50 ng/mL) for 10 min before lysis. Whole cell lysates were probed for the tyrosine phosphorylated form of STAT6. EGF treatment potently phosphorylated STAT6 on Y645 in both cell lines. STAT6 was also basally phosphorylated at a lower level in the U-87MG but not the U-1242MG cells.

To determine whether EGFR signaling regulates STAT6 activity in our GBM cells, we treated U-1242MG and U-87MG cells with EGF (50 ng/mL) for 5 minutes, lysed the cells and assessed STAT6 tyrosine phosphorylation (pY645) by Western blotting. In both cell lines, stimulation with EGF resulted in robust tyrosine phosphorylation of STAT6, indicating that STAT6 is in fact activated by this signaling pathway (Figure 3). In addition, basal phosphorylation of STAT6 was observed in the U87MG cell line but not in U1242 cell line.

### shRNA silencing of STAT6 in U-1242MG and U-87MG cells

We employed a lentiviral delivery system to stably decrease expression of STAT6 in the U-1242MG and U-87MG cells. Cells were transduced with one of five unique shRNA sequences, and the resulting mixed cultures were screened for successful STAT6 knockdown by Western blot analysis. Each mixed culture was also examined for expression of STAT3, STAT5a and STAT5b (not shown) to avoid misleading results due to non-specific knockdown of these other STATs. There is a high degree of homology between members of the STAT family, and significant non-specific knockdown was observed in at least one sequence for each cell line. Those mixed cultures derived from sequences that resulted in efficient STAT6 knockdown in the absence of obvious off-target effects were chosen for dilution cloning. Individual cells were expanded into clonal colonies and again screened for stable STAT6 knockdown.

**Figure 4:**
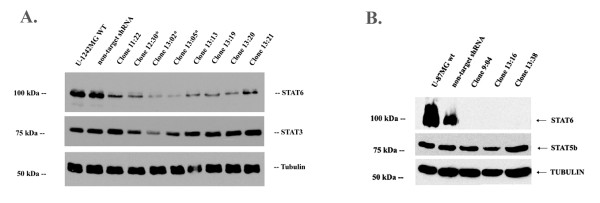
**STAT6 depletion by shRNA in two GBM cell lines**. U-1242MG and U-87MG cells were transduced with lentivirus containing either non-target shRNA or shRNA directed against STAT6, followed by selection with puromycin. Single-cell clones were screened for levels of STAT6. **Figure 4a**: Five representative clones are shown for U-1242MG. Expression of STATs 3 and 5b was not affected by shRNA against STAT6. Tubulin was the loading control. **Figure 4b**: Three representative clones are shown for U-87MG. Expression of STAT5b is not affected, and tubulin demonstrates even loading.

STAT6-deficient clones from each cell line were again screened for non-specific knockdown of other STATs. We chose to test for expression of STAT5b and STAT3 in U-87MG and U-1242MG, respectively, based on our previous results when screening the mixed cultures. In U-1242MG, for example, sequences 11 and 13 were the most effective and specific; there was virtually no knockdown of STAT5a or STAT5b, but a slight reduction in STAT3 expression was observed. Therefore, when selecting clones for functional studies, we chose to screen for STAT3 so that clones with normal STAT3 levels could be selected (Figure 4A). In U-87MG, STAT5b was most likely to be affected based on the mixed culture screens, possibly because STAT3 is expressed at very low levels in this cell line. We therefore chose to examine STAT5b expression as our specificity control for the individual clones (Figure 4B).

Control cells were also created for each cell line by infecting wild-type cells with a non-target shRNA in a lentiviral vector. As Figure 4 shows, these "non-target Control" groups had STAT6 levels similar to the wild-type cells while the knockdown clones showed a significant reduction in STAT6 protein expression. As seen in Figure 4A, there was a non-specific decrease in STAT3 in some of the stable STAT6 knockdown clones. These clones (labeled with *) were excluded from experiments. Given that in earlier screening experiments, different STAT6 shRNA sequences resulted in off-target knockdown of different STATs, this is most likely a result of high sequence homology between STATs and not a specific biological consequence of reduced STAT6 expression.

### shRNA-mediated gene silencing of STAT6 decreases proliferation of U-1242MG and U-87MG cells

**Figure 5:**
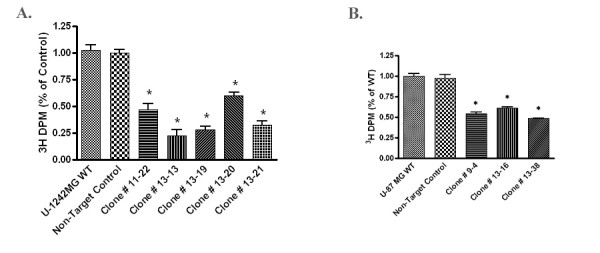
**shRNA-mediated STAT6 silencing blocks **^**3**^**H-thymidine uptake in U-1242MG and U-87MG GBM cells**. Equal numbers of cells were plated and allowed to grow in the presence of serum for 72 hours, then pulsed with ^3^H-thymidine prior to fixation, permeabilization and analysis. **Figure 5a**: Five representative STAT6 knockdown clones of the U-1242MG cell line showed a 40-70% decrease in^3^H-thymidine uptake compared to the non-target and wild-type controls. **Figure 5b**: Three representative U-87MG STAT6 knockdown clones had a reduction in ^3^H-thymidine uptake of 40-50% compared to controls. Error bars represent the S.E.M. and an asterisk (*) indicates statistically significant divergence from control as determined by student t-test. Experiments were repeated a minimum of three times, and results normalized against the non-target control. (P < 0.01)

In order to investigate the physiological importance of STAT6 in GBM, we measured ^3^H-thymidine incorporation into cellular DNA as an indicator of cell proliferation in wild-type cells and in the STAT6-deficient clones. As presented in Figure 5, the STAT6 knockdown clones exhibited significantly reduced ^3^H-thymidine uptake compared with the wild-type in both U-1242MG and U-87MG cells. In both cell lines, ^3^H-thymidine incorporation was reduced by 40% or more in all STAT6 knockdown clones, with some of the U-1242MG clones exhibiting up to a 70% decrease in uptake. As expected, the ^3^H-thymidine uptake of the non-target control was not significantly different from the wild-type in either cell line (Figure 5). These findings indicate that depletion of STAT6 from U-1242MG and U-87MG cells adversely affected their proliferative capacity, which suggests that one role of STAT-6 over-expression in GBM is to confer an enhanced growth rate and thereby, a selective advantage to individual tumor cells.

### STAT6 depletion by shRNA inhibits the invasion of glioma cells in vitro

GBMs are highly invasive tumors that often recur in remote brain areas less than a year following surgical resection. This high recurrence rate is in large part responsible for the dismal prognosis for GBM patients, as it makes surgical removal of the primary tumor mass an ineffective means of treatment. A better understanding of the mechanisms underlying the invasive behavior of GBM cells may provide clues on how to prevent or delay tumor recurrence in human patients.

In order to determine whether STAT6 is involved in mediating the invasiveness of GBM cells, we performed an *in vitro *invasion assay on wild type GBM cell lines, non-target control cells and the STAT6 knockdown clones. Equal numbers of cells were allowed to invade through a membrane coated with Type IV collagen substrate, toward a chemo attractant (1% FBS) for 8 hours. The invaded cells were fixed, stained and counted. We purposely chose a relatively short time point (8 h), in order to avoid a potential alteration of results by the differing cellular growth rates. The use of serum-free or very low-serum medium for U-1242MG and U-87MG, respectively, served as an additional control since neither cell line actively proliferates in the absence of serum.

**Figure 6:**
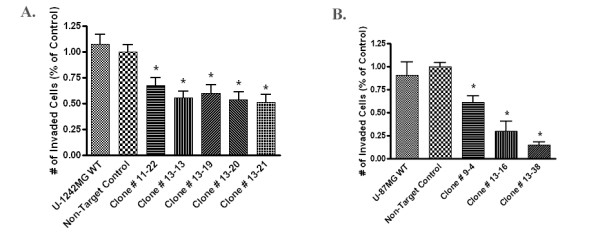
**Knockdown of STAT6 impairs invasion in U-1242MG and U-87MG**. Equal numbers of cells were seeded into the upper compartment of a transwell system and allowed to invade through a type IV collagen substrate for 8 hours. The invaded cells were then fixed and counted. **Figure 6a**: In U-1242MG, invasion was inhibited by 25-35% in the STAT6 knockdown clones compared to the non-target and wild type cells. **Figure 6b**: The effect was more dramatic in the STAT6-depleted U-87MG clones, which exhibited a decrease in invasion of 30-75%. Error bars represent the S.E.M. and an asterisk (*) indicates statistically significant divergence from control as determined by student t-test. Experiments were repeated a minimum of three times, and results normalized against the non-target control. (P < 0.01)

Figure 6 shows that the STAT6 knockdown cells were considerably less invasive than the wild type or non-target control cells (Figure 6A and 6B). This was the case for both cell lines, although the effect was more dramatic in U-87MG STAT6 knockdown clones, which exhibited a decrease in invasion of up to 80%, compared with wild type (Figure 6B). In U-1242MG, invasion was decreased by 25-35% following STAT6 depletion, while the non-target control cells invaded in similar numbers to the wild-type in both cell lines. The shRNA-silencing seemed to be more efficient in U-87 than in U1242, which may explain the invasion results. Importantly, there is no obvious correlation between individual clones that were least invasive and those with the greatest decrease in proliferation, suggesting that differences in cellular growth rates were not responsible for the results seen in the invasion assay.

### Changes in gene expression following STAT6 knockdown are cell-line dependent

While the apparent link between STAT6 expression and several aspects of GBM malignancy is intriguing, STAT6 itself is a transcription factor and as such, exerts its cellular effects by means of transcriptional targets. To our knowledge, STAT6 gene targets in GBM have not been described. We were therefore curious to see which genes would be differentially expressed following STAT6 knockdown in U-1242MG and U-87MG cells. In order to arrive at a comprehensive list of potential STAT6 target genes, we performed a microarray analysis on wild type U-1242MG and U-87MG cells as well as three STAT6 knockdown clones from each cell line. We utilized Human Genome U133-plus 2 Affymetrix oligonucleotide arrays, which contain approximately 56,400 transcripts of human genes or ESTs and thus provide a fairly complete overview of changes in gene expression. For each cell line, we compared the wild type to the group of the three clones; this way, the effects of any non-specific alterations in gene expression within individual clones on the overall comparison would be minimized. A complete list of genes whose expression was altered in the STAT6 knockdown clones compared to wild type can be seen in the additional files 1 and 2 (Additional file 1: Affymetrix Array_U87MG_3clones vs WT.xlsx; Additional file 2: Affymetrix Array_U1242MG_3clones vs WT and NT.xlsx) and additional file 3, which depicts a heat map of the data (Additional file 3: Affymetrix Array_U1242MG_U87MG_filtered clustering.png). Tables 2 and 3 show an abbreviated list of genes whose expression was the most dramatically decreased in the clones of U-1242MG and U-87MG cells, respectively. Notably, there is virtually no overlap between the genes affected by STAT6 knockdown in the two cell lines; it appears that STAT6 targets an entirely different set of genes in U-1242MG and U-87MG (Tables 2 and 3).

**Table 2:** Genes with reduced expression levels following STAT6 knockdown in U-1242MG cells.

U-1242MG	
**Gene**	**Fold reduction in STAT6 clones**

**ATP-binding cassette, sub-family D (ALD), member 3**	**1.58**

**Vav 2 oncogene**	**1.59**

**DKFZP566K1924 protein**	**1.6**

**Endothelial differentiation, spingolipid G-protein-coupled receptor, 3**	**1.63**

**Nudix (nucleoside diphosphate linked moiety X)-type motif 1**	**1.67**

**Adipose differentiation-related protein**	**1.71**

**Laminin, gamma 2**	**1.93**

**GATA binding protein 2**	**1.93**

**Plasminogen activator, urokinase**	**1.98**

**MAD, mothers against decapentaplegic homolog 6 (Drosophila)**	**2.17**

**Interferon-induced protein 44**	**2.35**

**2',5'-oligoadenylate synthetase 1, 40/46kDa**	**2.38**

**Heparan sulfate 6-O-sulfotransferase 1**	**2.41**

**DEAD/H (Asp-Glu-Ala-Asp/His) box polypeptide**	**2.43**

**KIAA1026 protein**	**2.59**

**Interferon-induced protein with tetratricopeptide repeats 4**	**2.6**

**Interleukin 18 (interferon-gamma-inducing factor)**	**2.93**

**Solute carrier family 1 (neuronal/epithelial high affinity glutamate transporter, system Xaq), member 1**	**3.65**

**Stratifin**	**4.75**

**Viperin**	**4.76**

**Table 3:** Genes with reduced expression levels following STAT6 knockdown in U-87MG cells.

U-87MG	
**Gene**	**Fold reduction in STAT6 clones**

**Matrix metalloproteinase 1 (interstitial collagenase)**	**2.68**

**Forming-like 2**	**2.68**

**Transmembrane gamma-carboxyglutamic acid protein4**	**2.69**

**Interleukin 1 family, member 8 (eta)**	**2.77**

**Gamma-aminobutyric acid (GABA) A receptor, beta 1**	**2.8**

**Rho guanine nucleotide exchange factor (GEF) 3**	**2.92**

**Protocadherin 17**	**2.97**

**Aldo-keto reductase family 1, member C1 (dihydrodiol dehydrogenase 1; 20-alpha (3-alpha)-hydroxysteroid dehydrogenase**	**2.99**

**Purinergic receptor P2Y, G-protein coupled, 8**	**3**

**Major histocompatibility complex, class II, DQ alpha 1**	**3.09**

**Thymus high mobility group box protein TOX**	**3.14**

**Melanoma antigen, family E, 1, cancer/testis specific**	**3.17**

**SRY (sex determining region Y)-box 7**	**3.22**

**Lung type-I cell membrane-associated glycoprotein (PDPN/podoplanin)**	**3.4**

**Interleukin 1 receptor antagonist**	**3.46**

**Hemicentin**	**3.54**

**Aquaporin 9**	**3.76**

**Ras-related GFP binding D**	**3.92**

**Neuromedin U**	**4.76**

**Guinolinate phosphoribosyltransferase (nicotinate-nucleotide pyrophosphorylase (carboxylating))**	**4.97**

**CD24 antigen (small cell lung carcinoma cluster 4 antigen)**	**5.1**

**Putative lymphocyte G0/G1 switch gene**	**5.72**

**Chemokine (C-X-C motif) ligand 5**	**5.77**

### STAT6 gene expression correlates with survival in human glioma patients

**Figure 7:**
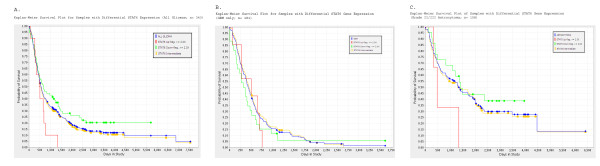
**STAT6 expression correlates with survival in human glioma patients**. The Rembrandt database was used to perform a Kaplan-Meier survival analysis of glioma patients with differential STAT6 expression. Up- and down-regulation were defined as a two-fold or greater deviation from intermediate expression. **Figure 7a**: Among all glioma patients (n= 343) there was a significant difference in survival (p < 0.05) between patients with increased vs. decreased STAT6 gene expression. **Figure 7b**: In the subset of patients with Grade IV tumors/GBM (n= 181), there was a clear trend towards a similar correlation between STAT6 expression and survival; however statistical significance was not reached. **Figure 7c**: In patients with Grade II/III astrocytoma (n= 105), a similar trend was observed.

Based on our *in vitro *data (Figures 5 and 6) relating STAT6 expression to increased GBM growth and invasion, we hypothesized that increased STAT6 expression would also correlate with a worse prognosis in glioma patients. To test this theory, we took advantage of the publicly available patient data in the NCI Repository for Molecular Brain Neoplasia Data (REMBRANDT) database [1]. Using microarray-based gene expression data and associated clinical reports, we generated a Kaplan-Meier survival curve based on differential STAT6 expression among 343 glioma patients (Figure 7A). They included patients with GBMs (181), grade II/III astrocytomas (105), grade II/III oligodendrogliomas (50), and mixed tumors (7). Up-and down-regulation were defined as a two-fold increase or decrease in STAT6 expression, respectively, compared to the mean expression level within the given data set. Based on these criteria, STAT6 was up-regulated in ten patients, down-regulated in 72 and expressed at an intermediate level in the remaining 261 patients.

The graph shows a trend toward increased survival times for patients with decreased STAT6 expression, as well as a worse prognosis in cases of STAT6 up-regulation (Figure 7A). However, statistical significance was only reached when comparing survival in these two extreme patient populations (p = 0.046 between patients with STAT6 up- versus down-regulation), although other comparisons would likely gain statistical significance if the sample size were increased. Figures 7B and 7C show the same analysis performed on GBM patients and Grade II/III astrocytoma patients, respectively. Statistical significance is not reached in these patient populations, possibly due to insufficient sample size. There is however a trend correlating longer survival times with lower STAT6 expression in both patient subsets.

## Discussion

STAT proteins were originally identified as signaling molecules involved in interferon-dependent cellular responses [45,46]. They were soon recognized as important mediators of cytokine production [47-50], particularly as it pertains to development and the immune response [9].

More recently, it has been demonstrated that STATs 3 and 5 are found in a significant percentage of human malignancies, where they contribute to growth, survival, and metastasis of cancer cells [19,51-56]. STAT1 on the other hand renders malignant cells more susceptible to apoptotic stimuli [57,58]. The remaining STAT family members, namely STATs 2, 4, and 6, are still regarded as having limited importance in cancer biology [9].

In this report, we have shown robust STAT6 protein expression in two GBM cell lines, and further demonstrated that STAT6 expression in these cells positively correlated with their rate of proliferation, as well as their invasive capacity. These findings are in agreement with reports by others, which suggest that STAT6 is involved in regulating the proliferation of hematopoietic cells [34,59], fibroblasts [60] and vascular smooth muscle cells [61], and that it is involved in facilitating metastasis of colon cancer cells [28] as well as migration of prostate cancer cells [62]. Suggested mechanisms through which STAT6 enhances cell proliferation include up-regulation of Cdk6, which facilitates cell cycle progression, and Myc, which up-regulates additional components of the cell cycle [58]. The exact mechanism by which STAT6 regulates proliferation and invasion in GBM remains to be explored; however, our microarray analysis did identify several potential STAT6 target genes which promote these behaviors in other malignancies. For instance, the expression of matrix metalloproteinase 1 (MMP-1) and urokinase plasminogen activator (uPa) is reduced in the STAT6 knockdown clones of U-87MG and U-1242MG cells, respectively (Tables 2 and 3). Both MMP-1 and uPA have demonstrated roles in facilitating invasion and metastasis of cancer cells, presumably via degradation of the basement membrane surrounding the tumor [63-66]. Lung type I cell membrane-associated glycoprotein, also known as podoplanin, has been implicated in promoting invasion of astrocytomas [67]; its expression also declines following STAT6 knockdown in U-87MG (Table 3).

We validated the relevance of our *in vitro *findings by assessing STAT6 expression in human patient astrocytoma specimens of different malignancy. STAT6 was detected by IHC in the majority of human astrocytoma specimens ranging from Grade I (pilocytic astrocytoma) to Grade IV (GBM), but notably not in any normal brain sections. In the patient tumors, STAT6 was localized almost exclusively in the nucleus, which suggests that it existed in a phosphorylated (active) state- quite unlike STAT5, which others have shown to be located primarily in the cytoplasm [19]. The implications of this latest finding have yet to be explored in detail.

The lack of correlation between STAT6 expression and tumor grade suggests that STAT6 is involved early in tumor development but is not dispensable later on as the tumor progresses. It is possible that STAT6 performs similar functions in low- and high-grade tumors; promotion of cell proliferation for example would be beneficial to tumors in any stage of development. On the other hand, the apparent contribution of STAT6 to the invasive capabilities of GBM cells contradicts such a model, since invasion is exclusively a hallmark of high-grade tumors. A likely scenario is that additional pro-invasive factors must be present in order for STAT6 target genes to perform this function. It is also conceivable that STAT6 induces expression of a different subset of transcriptional targets based on the availability of transcriptional co-factors, which likely varies between low- and high-grade gliomas. In fact, our microarray analysis demonstrated that STAT6 appears to have non-identical target genes in two different GBM cell lines, suggesting that even among Grade IV/GBM tumors, its primary downstream effectors may differ considerably. These results highlight the already well-documented heterogeneity of GBMs, and underscore the importance of multi-target therapeutic approaches.

Lastly, we showed the clinical and potentially prognostic significance of STAT6 up- and down-regulation in glioma patients by demonstrating that STAT6 expression inversely correlates with overall survival. In a Kaplan-Meier survival analysis of 343 glioma patient datasets obtained from Rembrandt [1], lower STAT6 expression levels were indicative of a more favorable prognosis compared to patients with intermediate or high STAT6 expression. When the same analysis was performed on data for GBM patients and Grade II/III astrocytoma patients separately, a non-significant trend showed a similar correlation between increased STAT6 expression and shorter survival times, suggesting that the initial findings were not biased by differential expression in high- versus low-grade tumors. These findings are in perfect agreement with our earlier observations that STAT6 contributes to a more malignant phenotype by promoting GBM cell proliferation and invasion.

The results described here support other works advocating an increasingly complex regulatory role for STAT6 in the context of cancer. For example, reports in the literature describe anti-apoptotic effects of STAT6 in primary B cells [68], Hodgkin lymphoma cells [58] and colon cancer cells [35]. Others have demonstrated the contribution of STAT6 to the suppression of an effective anti-tumor immune response in STAT6 ^-/- ^mice [69-74]. The combination of our findings and published reports by other groups thus suggests multiple functions for STAT6 in the promotion and/or maintenance of tumors, including enhancement of proliferation, invasion, survival and immune evasion. Importantly, in our study the effects of STAT6 expression on the behavior of tumor cells appear to depend on its expression within the tumor cells themselves, whereas aforementioned reports attributed improved immunological responses in STAT6^-/- ^animals to STAT6 depletion in cells comprising the tumor microenvironment [69-74]. This suggests the possibility of synergistic benefits in response to global- rather than tumor specific- inhibition of STAT6 *in vivo*.

Immuno-therapeutic approaches to GBM treatment are generally seen as promising but thus far have been only moderately effective [3,75,76]. The limited success of GBM cancer vaccine trials- and cancer vaccine trials in general- can be at least in part attributed to the fact that many tumors, including GBM, can actively suppress an effective vaccine-induced immune response by releasing specific cytokines into the tumor microenvironment, thereby preventing the appropriate activation, differentiation and/or tumor infiltration of CD8^+ ^T cells [3,69]. Others have shown that STAT6 is a critical inhibitory regulator of CD8^+ ^T cell activation and appropriate tissue infiltration *in vivo *[70,73]. Accordingly, STAT6 knock-out mice (STAT6 ^-/- ^mice) have markedly enhanced anti-tumor immunity, as demonstrated by a reduced incidence of spontaneous primary tumors, significantly slower growth of xenografts, a drastically reduced incidence of metastases, and a very low recurrence rate of surgically excised aggressive primary tumors when compared with STAT6 ^+/+ ^mice [71-76]. Importantly, the relative resistance of the STAT6 ^-/- ^mice to xenograft tumors suggests that the enhanced anti-tumor immunity observed in these animals is a not a consequence of STAT6 depletion in the tumor cells, but rather results from its loss within the host tumor microenvironment. These findings, combined with our data demonstrating the contribution of STAT6 to the malignancy of tumor cells via promotion of proliferation and invasion, raise the interesting possibility that STAT6 may perform tumor-supportive roles in both the tumor itself and in the surrounding stromal compartment. This would suggest that the potential benefits of STAT6 inhibition could be two-fold: enhanced anti-tumor immunity combined with growth inhibition and decreased invasive potential of the tumor cells. Given that GBM recurrence after surgical resection is virtually 100%, a combinatorial treatment targeting tumor cells while also stimulating host immunity has potential to result in improved treatment outcomes.

## Conclusions

In conclusion, based on the findings in this paper and reports in the literature, it appears that targeting STAT6 could be a promising new approach to GBM treatment, which would potentially accomplish dual goals: it would act on the tumor directly to slow its growth and inhibit invasion into surrounding tissues, while simultaneously enhancing the patient's own immune response against the tumor. Given that GBM is a particularly aggressive malignancy that has been exceptionally resistant to virtually all attempts at treatment, a new approach targeting the tumor in multiple ways may turn out to be more effective than currently available therapies.

## Competing interests

The authors declare that they have no competing interests.

## Authors' contributions

MSL performed the tissue microarray study. BCM performed all other experiments, data analysis and drafted the manuscript. JLO contributed to data collection and initial conception of the study. CMS helped with experimental design, provided intellectual expertise throughout the process, and critically reviewed the manuscript. IMH conceived, designed and supervised experimental work and manuscript editing. All authors read and approved the final manuscript.

## Pre-publication history

The pre-publication history for this paper can be accessed here:

http://www.biomedcentral.com/1471-2407/11/184/prepub

## Supplementary Material

Additional file 1 **Gene clustering map **Visual representation of changes in gene expression following STAT6 silencing in U-87MG and U-1242MG GBM cells. Click here for file

Additional file 2 **Genes with altered expression levels in U-87MG STAT6 knockdown clones **compared to wild type. This file includes microarray data for all genes whose expression was increased or decreased at least 1.5 fold in the grouping of the three U-87MG STAT6 clones compared to the wild type control. Click here for file

Additional file 3 **Genes with altered expression levels in U-1242MG STAT6 knockdown clone compared to wild type**. This file includes microarray data for all genes whose expression was increased or decreased at least 1.5 fold in the grouping of the three U-1242MG STAT6 clones compared to the grouping of the wild type and non-target controls. Click here for file

## References

[B1] National Cancer Institute REMBRANDT home page 2005 http://rembrandt.nci.nih.gov Accessed 2010 October 1^st^

[B2] Kleihues P Burger PC Aldape KD Brat DJ Biernat W Bigner DD Nakazato Y Plate KH Giangespero F von Deimling A Ohgaki H Cavenee WK Louis DN, Ohgaki H, Wiestler OD, Cavanee WK WHO Classification of Tumors of the Central Nervous System 2007 IARC Press: Lyon 33 49

[B3] Wheeler CJ Black KL Liu G Mazer M Zhang X Pepkowitz S Goldfinger D Ng H Irvin D Yu JS Vaccination Elicits Correlated Immune and Clinical Responses in Glioblastoma Multiforme Patients Cancer Research 2008 68 5955 5964 10.1158/0008-5472.CAN-07-5973 18632651

[B4] Barcellos-Hoff MH Newcomb EW Zagzag D Narayana A Therapeutic targets in malignant glioblastoma microenvironment Semin.Radiat.Oncol 2009 19 163 170 10.1016/j.semradonc.2009.02.004 19464631 PMC3538148

[B5] Reardon DA Wen PY Therapeutic Advances in the Treatment of Glioblastoma: Rationale and Potential Role of Targeted Agents The Oncologist 2006 11 152 164 10.1634/theoncologist.11-2-152 16476836

[B6] Shin-ei N El-Jawahri A Patel D Lautenschlaeger T Siedow M Chakravarti A Molecular Advances of Brain Tumors in Ratiation Oncology Seminars in Radiation Oncology 2009 19 171 178 10.1016/j.semradonc.2009.02.005 19464632

[B7] Stupp R Mason WP van den Bent MJ Weller M Fisher B Taphoorn MJ Belanger K Brandes AA Marosi C Bogdahn U Curschmann J Janzer RC Ludwin SK Gorlia T Allgeier A Lacombe D Cairncross JG Eisenhauer E Mirimanoff RO Radiotherapy plus concomitant and adjuvant temozolomide for glioblastoma N Engl J Med 2005 352 987 996 10.1056/NEJMoa043330 15758009

[B8] Haura EB Zheng Z Song L Cantor A Bepler G Activated epidermal growth factor receptor-Stat-3 signaling promotes tumor survival in vivo in non-small cell lung cancer Clin.Cancer Res 2005 11 8288 8294 10.1158/1078-0432.CCR-05-0827 16322287

[B9] Bowman T Garcia R Turkson J Jove R STATs in oncogenesis Oncogene 2000 19 2474 2488 10.1038/sj.onc.1203527 10851046

[B10] Grandis JR Pietenpol JA Greenberger JS Pelroy RA Mohla S Head and neck cancer: meeting summary and research opportunities Cancer Res 2004 64 8126 8129 10.1158/0008-5472.CAN-04-2445 15520225

[B11] Buettner R Mora LB Jove R Activated STAT signaling in human tumors provides novel molecular targets for therapeutic intervention Clin.Cancer Res 2002 8 945 954 11948098

[B12] Atreya R Neurath MF Signaling molecules: the pathogenic role of the IL-6/STAT-3 trans signaling pathway in intestinal inflammation and in colonic cancer Curr Drug Targets 2008 9 369 74 10.2174/138945008784221116 18473764

[B13] Corvinus FM Orth C Moriggl R Tsareva SA Wagner S Pfitzner EB Baus D Kaufmann R Huber LA Zatloukal K Beug H Ohlschlager P Schutz A Halbhuber KJ Friedrich Persistent STAT3 activation in colon cancer is associated with enhanced cell proliferation and tumor growth Neoplasia 2005 7 545 555 10.1593/neo.04571 16036105 PMC1501283

[B14] Migone TS Lin JX Cereseto A Mulloy JC O'Shea JJ Franchini G Leonard WJ Constitutively activated Jak-STAT pathway in T cells transformed with HTLV-I Science 1995 269 79081 10.1126/science.7604283 7604283

[B15] Zhang Q Nowak I Vonderheid EC Rook AH Kadin ME Nowell PC Activation of Jak/STAT proteins involved in signal transduction pathway mediated by receptor for interleukin 2 in malignant T lymphocytes derived from cutaneous anaplastic large T-cell lymphoma and Sezary syndrome Proc Natl Acad Sci 1996 93 9148 9153 10.1073/pnas.93.17.9148 8799169 PMC38610

[B16] Schaefer LK Ren Z Fuller GN Schaefer TS Constitutive activation of Stat3alpha in brain tumors: localization to tumor endothelial cells and activation by the endothelial tyrosine kinase receptor (VEGFR-2) Oncogene 2002 27 2058 65 10.1038/sj.onc.1205263 11960378

[B17] Grandis JR Sok JC Signaling through the epidermal growth factor receptor during the development of malignancy Pharmacol Ther 2004 102 37 46 10.1016/j.pharmthera.2004.01.002 15056497

[B18] Konnikova L Kotecki M Kruger MM Cochran BH Knockdown of STAT3 expression by RNAi induces apoptosis in astrocytoma cells BMC Cancer 2003 3 23 10.1186/1471-2407-3-23 13678425 PMC212316

[B19] Liang QC Xiong H Zhao ZW Jia D Li WX Qin HZ Deng JP Gao L Zhang H Gao GD Inhibition of transcription factor STAT5b suppresses proliferation, induces G1 cell cycle arrest and reduces tumor cell invasion in human glioblastoma multiforme cells Cancer Letters 2009 273 164 171 10.1016/j.canlet.2008.08.011 18793823

[B20] Park OK Schaefer TS Nathans D In vitro activation of Stat3 by epidermal growth factor receptor kinase Proc Natl Acad Sci 1996 93 13704 8 10.1073/pnas.93.24.13704 8942998 PMC19397

[B21] Decker T Kovarik P Transcription factor activity of STAT proteins: structural requirements and regulation by phosphorylation and interacting proteins Cellular and Molecular Life Sciences 1999 55 1535 1546 10.1007/s000180050393 10526571 PMC11146901

[B22] Mui A The role of STATs in proliferation, differentiation, and apoptosis Cellular and Molecular Life Sciences 1999 55 1547 1558 10.1007/s000180050394 10526572 PMC11146798

[B23] Grad JM Zeng XR Boise LH Regulation of Bcl-xL: a little bit of this and a little bit of STAT Curr Opin Oncol 2000 12 543 9 10.1097/00001622-200011000-00006 11085453

[B24] Bromberg JF Wrzeszczynska MH Devgan G Zhao Y Pestell RG Albanese C Darnell JE Jr Stat3 as an oncogene Cell 1999 6 295 303 10.1016/s0092-8674(00)81959-5 10458605

[B25] deGroot RP Raaijmakers JA Lammers JW Koenderman L STAT5-Dependent CyclinD1 and Bcl-xL expression in Bcr-Abl-transformed cells Mol Cell Biol Res Commun 2000 3 299 305 10.1006/mcbr.2000.0231 10964754

[B26] Dang CV Resar LM Emison E Kim S Li Q Prescott JE Wonsey D Zeller K Function of the c-Myc Oncogenic Transcription Factor Experimental Cell Research 1999 253 63 77 10.1006/excr.1999.4686 10579912

[B27] Kiuchi N Nakajima K Ichiba M Fukada T Narimatsu M Mizuno K Hibi M Hirano T STAT3 is required for the gp130-mediated full activation of the c-myc gene J exp Med 1999 189 63 73 10.1084/jem.189.1.63 9874564 PMC1887683

[B28] Ni Z Lou W Lee SO Dhir R DeMiguel F Grandis JR Gao AC Selective activation of members of the signal transducers and activators of transcription family in prostate carcinoma J Urol 2002 167 1859 1862 10.1016/S0022-5347(05)65249-4 11912448

[B29] Li BH Yang XZ Li PD Yuan Q Liu XH Yuan J Zhang WJ IL-4/Stat6 activities correlate with apoptosis and metastasis in colon cancer cells Biochemical and Biophysical Research Communications 2008 369 554 560 10.1016/j.bbrc.2008.02.052 18294957

[B30] Benekli M Baer MR Baumann H Wetzler M Signal transducer and activator of transcription proteins in leukemias Blood 2003 101 2940 2954 10.1182/blood-2002-04-1204 12480704

[B31] Skinnider BF Elia AJ Gascoyne RD Patterson B Trumper L Kapp U Mak TW Signal transducer and activator of transcription 6 is frequently activated in Hodgkin and Reed-Sternberg cells of Hodgkin lymphoma Blood 2002 99 618 626 10.1182/blood.V99.2.618 11781246

[B32] Guiter C Dusanter-Fourt I Copie-Bergman C Boulland ML Le Gouvello S Gaulard P Leroy K Castellano F Constitutive STAT6 activation in primary mediastinal large B-cell lymphoma Blood 2004 104 543 549 10.1182/blood-2003-10-3545 15044251

[B33] Melzner I Bucur AJ Bruderlein S Dorsch K Hasel C Barth TF Leithauser F Moller P Biallelic mutation of SOCS-1 impairs JAK2 degradation and sustains phospho-JAK2 action in the MedB-1 mediastinal lymphoma line Blood 2005 105 2535 2542 10.1182/blood-2004-09-3701 15572583

[B34] Bruns HA Kaplan MH The role of constitutively active Stat6 in leukemia and lymphoma Critical Reviews in Oncology/Hematology 2006 57 245 253 10.1016/j.critrevonc.2005.08.005 16213149

[B35] Zhang M Zhou Y Zhang W Zhang X Pan Q Ji XM Luo ZG Wu JP Apoptosis induced by short hairpin RNA-mediated STAT6 gene silencing in human colon cancer cells Chinese Medical Journal 2006 119 801 808 16732981

[B36] Hussaini IM Karns LR Vinton G Carpenter JE Redpath GT Sando JJ Phorbol 12-myristate 13-acetate induces protein kinase ceta-specific proliferative response in astrocytic tumor cells J.Biol.Chem 2000 275 22348 22354 10.1074/jbc.M003203200 10806212

[B37] Amos S Mut M diPierro CG Carpenter JE Xiao A Kohutek ZA Redpath GT Zhao Y Wang J Shaffrey ME Hussaini IM Protein kinase C-alpha-mediated regulation of low-density lipoprotein receptor related protein and urokinase increases astrocytoma invasion Cancer Research 2007 67 10241 10251 10.1158/0008-5472.CAN-07-0030 17974965 PMC2386949

[B38] Gallagher J Howlin J McCarthy C Murphy EP Bresnihan B FitzGerald O Godson C Brady HR Martin F Identification of Naf1/ABIN-1 among TNF-alpha-induced expressed genes in human synoviocytes using oligonucleaotide microarrays FEBS Lett 2003 551 8 12 10.1016/S0014-5793(03)00823-8 12965196

[B39] Li C Wong WH Model-based analysis of oligonucleotide arrays: Expression index computation and outlier detection Proc Natl Acad Sci 2001 in press 10.1073/pnas.011404098 PMC14539 11134512

[B40] Brantley EC Benveniste EN Signal Transducer and Activator of Transcription-3: A Molecular Hub for Signaling Pathways in Gliomas Mol Cancer Res 2008 6 675 684 10.1158/1541-7786.MCR-07-2180 18505913 PMC3886801

[B41] Iwamaru A Szymanski S Iwado E Aoki H Yokoyama T Fokt I Hess K Conrad C Madden T Sawaya R Kondo S Priebe W Kondo Y A novel inhibitor of the STAT3 pathway induces apoptosis in malignant glioma cells both in vitro and in vivo Oncogene 2006 26 2435 2444 17043651 10.1038/sj.onc.1210031

[B42] Gu J Li G Sun T Su Y Zhang X Shen J Tian Z Zhang J Blockage of the STAT3 signaling pathway with a decoy oligonucleotide suppresses growth of human malignant glioma cells J Neurooncol 2008 89 9 17 10.1007/s11060-008-9590-9 18415045

[B43] Hussain SF Kong LY Jordan J Conrad C Madden T Fokt I Priebe W Heimberger AB A novel small molecule inhibitor of signal transducers and activators of transcription 3 reverses immune tolerance in malignant glioma patients Cancer Research 2007 67 9630 9636 10.1158/0008-5472.CAN-07-1243 17942891

[B44] Rahaman SO Sharma P Harbor PC Aman MJ Vogelbaum MA Haque SJ IL-13Rα2, a Decoy Receptor for IL-13 Acts As an Inhibitor of IL-4-dependent Signal Transduction in Glioblastoma Cells Cancer Research 2002 62 1103 1109 11861389

[B45] Darnell JE STATs and Gene Regulation Science 1997 277 1630 1635 10.1126/science.277.5332.1630 9287210

[B46] Stark GR How cells respond to interferons revisited: From early history to current complexity Cytokine Growth Factor Rev 2007 18 419 423 10.1016/j.cytogfr.2007.06.013 17683974 PMC2081984

[B47] Heim MH The Jak-Stat pathway: cytokine signaling from the receptor to the nucleus J Recept Signal Transduct Res 1999 19 75 120 10.3109/10799899909036638 10071751

[B48] Liu KD Gaffen SL Goldsmith MA JAK/STAT signaling by cytokine receptors Current Opinion in Immunology 1998 10 271 278 10.1016/S0952-7915(98)80165-9 9638363

[B49] Ihle JM The Stat family in cytokine signaling Current Opinion in Cell Biology 2001 13 211 217 10.1016/S0955-0674(00)00199-X 11248555

[B50] Guo L Wei G Zhu J Liao W Leonard WJ Zhao K Paul W IL-1 family members and STAT activators induce cytokine production by Th2, Th17, and Th1 cells PNAS 2009 106 13463 13468 10.1073/pnas.0906988106 19666510 PMC2726336

[B51] Turkson J Bowman T Garcia R Caldenhoven E De Groot RP Jove R Stat3 activation by Src induces specific gene regulation and is required for cell transformation Mol Cell Biol 1998 18 2545 52 9566874 10.1128/mcb.18.5.2545 PMC110634

[B52] Pedranzini L Leitch A Bromberg J Stat3 is required for the development of skin cancer J Clin Invest 2004 114 619 22 15343379 10.1172/JCI22800 PMC514594

[B53] Devarajan E Huang S STAT3 as a central regulator of tumor metastases Curr Mol Med 2009 9 626 33 10.2174/156652409788488720 19601811

[B54] Melnikova VO Bar-Eli M Transcriptional control of the melanoma malignant phenotype Cancer Biol Ther 2008 7 997 1003 10.4161/cbt.7.7.6535 18698165

[B55] Wagner KU Rui H Jak2/Stat5 signaling in mammogenesis, breast cancer initiation and progression J Mammary Gland Biol Neoplasia 2008 13 93 103 10.1007/s10911-008-9062-z 18228120

[B56] Tan SH Nevalainen MT Signal transducer and activator of transcription 5A/B in prostate and breast cancers Endocr Relat Cancer 2008 15 367 90 10.1677/ERC-08-0013 18508994 PMC6036917

[B57] Townsend PA Cragg MS Davidson SM McCormick J Barry S Lawrence KM Knight RA Hubank M Chen PL Latchman DS Stephanou A STAT-1 facilitates the ATM activated checkpoint pathway following DNA damage J Cell Sci 2005 118 1629 39 10.1242/jcs.01728 15784679

[B58] Baus D Nonnenmacher F Jankowski S Döring C Bräutigam C Frank M Hansmann ML Pfitzner E STAT6 and STAT1 are essential antagonistic regulators of cell survival in classical Hodgkin lymphoma cell line Leukemia 2009 1 9 10.1038/leu.2009.103 19440213

[B59] Soon L Flechner L Gutkind JS Wang LH Baserga R Pierce JH Li W Insulin-like growth factor I synergizes with interleukin 4 for hematopoietic cell proliferation independent of insulin receptor substrate expression Mol Cell Biol 1999 19 3816 28 10207105 10.1128/mcb.19.5.3816 PMC84225

[B60] Kriebel P Patel BK Nelson SA Grusby MJ LaRochelle WJ Consequences of Stat6 deletion on Sis/PDGF- and IL-4-induced proliferation and transcriptional activation in murine fibroblasts Oncogene 1999 18 7294 302 10.1038/sj.onc.1203148 10602484

[B61] Baetta R Soma M De-Fraja C Comparato C Terruzi C Magrassi L Cattaneo E Upregulation and activation of Stat6 precede vascular smooth muscle cell proliferation in carotid artery injury model Arterioscler Thromb Vasc Biol 2000 20 931 9 10764656 10.1161/01.atv.20.4.931

[B62] Das S Roth CP Wasson LM Vishwanatha JK Signal transducer and activator of transcription -6 (STAT6) is a constitutively expressed survival factor in human prostate cancer Prostate 2007 67 1550 64 10.1002/pros.20640 17705178

[B63] Wang FQ Fisher J Fishman DA MMP-1-PAR1 axis mediates LPA-induced epithelial ovarian cancer (EOC) invasion Gynecol Oncol 2011 120 247 55 10.1016/j.ygyno.2010.10.032 21093894

[B64] Amos S Redpath GT Dipierro CG Carpenter JE Hussaini IM Epidermal growth factor receptor-mediated regulation of urokinase plasminogen activator expression and glioblastoma invasion via C-SRC/MAPK/AP-1 signaling pathways J Neuropathol Exp Neurol 2010 69 582 92 10.1097/NEN.0b013e3181e008fe 20467333

[B65] Takahashi S Yamada-Okabe H Hamada K Ohta S Kawase T Yoshida K Toda M Downregulation of uPARAP mediates cytoskeletal rearrangements and decreases invasion and migration properties in glioma cells J Neurooncol 2010 in press 10.1007/s11060-010-0398-z 20845060

[B66] Pei D Matrix metalloproteinases target protease-activated receptors on the tumor cell surface Cancer Cell 2005 7 207 8 10.1016/j.ccr.2005.02.011 15766657

[B67] Cortez MA Nicoloso MS Shimizu M Rossi S Gopisetty G Molina JR Carlotti C Jr Tirapelli D Neder L Brassesco MS Scrideli CA Tone LG Georgescu MM Zhang W Puduvalli V Calin GA miR-29b and miR-125a regulate podoplanin and suppress invasion in glioblastoma Genes Chromosomes Cancer 2010 49 981 90 10.1002/gcc.20808 20665731 PMC5559292

[B68] Wurster AL Rodgers VL White MF Rothstein TL Grusby MJ Interleukin-4-mediated protection of primary B cells from apoptosis through Stat6-dependent up-regulation of Bcl-xL J Biol Chem 2002 277 27169 75 10.1074/jbc.M201207200 12023955

[B69] Ostrand-Rosenberg S Clements VK Terabe M Park JM Berzofsky JA Dissanayake SK Resistance to Metastatic Disease in STAT6-Deficient Mice Requires Hemopoietic and Nonhemopoietic Cells and Is IFN-γ Dependent The Journal of Immunology 2002 169 5796 5804 12421960 10.4049/jimmunol.169.10.5796

[B70] Sinha P Clements VK Miller S Ostrand-Rosenberg S Tumor immunity: a balancing act between T cell activation, macrophage activation and tumor-induced immune suppression Cancer Immunol Immunother 2005 54 1137 1142 10.1007/s00262-005-0703-4 15877228 PMC11032820

[B71] Ostrand-Rosenberg S Grusby MJ Clements VK Cutting edge: STAT6-deficient mice have enhanced tumor immunity to primary and metastatic mammary carcinoma The Journal of Immunology 2000 165 6015 19 11086031 10.4049/jimmunol.165.11.6015

[B72] Jensen SM Meijer SL Kurt RA Urba WJ Hu HM Fox BA Regression of a mammary adenocarcinoma in STAT6-/- mice is dependent on the presence of STAT6-reactive T cells The Journal of Immunology 2003 170 2014 21 12574371 10.4049/jimmunol.170.4.2014

[B73] Terabe M Matsui S Noben-Trauth N Chen H Watson C Donaldson DD Carbone DP Paul WE Berzofsky JA NKT cell-mediated repression of tumor immunosurveillance by IL-13 and the IL-4R-STAT6 pathway Nature Immunology 2000 1 515 520 10.1038/82771 11101874

[B74] Kacha AK Fallarino F Markiewicz MA Gajewski TF Cutting edge: spontaneous rejection of poorly immunogenic P1.HTR tumors by Stat6-deficient mice The Journal of Immunology 2000 165 6024 28 11086033 10.4049/jimmunol.165.11.6024

[B75] Wheeler CJ Black KL DCVax-Brain and DC vaccines in the treatment of GBM Expert Opin Investig Drugs 2009 18 509 519 10.1517/13543780902841951 19335279

[B76] Yamanaka R Cell- and peptide-based immunotherapeutic approaches for glioma Trends in Molecular Medicine 2008 14 228 235 10.1016/j.molmed.2008.03.003 18403264

